# Supplementary material for: Brucella activates the host RIDD pathway to subvert BLOS1-directed immune defense
Source: eLife. 2022 May 19;11:e73625. doi: 10.7554/eLife.73625 (PMC9119680; doi:10.7554/eLife.73625)
Supplement: Supplementary file 1. — Recombinant plasmids constructed in this work. [file elife-73625-supp1.docx]

**Table S1. Recombinant plasmids constructed in this work.**

| Plasmids | Origin/Source | Identifier |
| --- | --- | --- |
| pLentiGUIDE-Puro-GFP2 | Gift from the Watson/Patrick Lab | N/A |
| pLentiGUIDE-Puro | ([Sanjana *et al*, 2014](#_ENREF_3)) | Addgene plasmid #52963 |
| pLentiGUIDE-Puro-Blos1-gRNA | This Paper | N/A |
| pCR™ 2.1-TOPO | Thermo Fisher | 451641 |
| pCR™ 2.1-TOPO-WT-Blos1 | This Paper | N/A |
| pCR™ 2.1-TOPO-Blos1-G449T | This Paper | N/A |
| pE2n | ([Dubin *et al*, 2008](#_ENREF_2)) | Addgene plasmid #17456 |
| pE2n-WT-Blos1 | This Paper | N/A |
| pE2n-Blos1-G449T | This Paper | N/A |
| pLenti-CMV-Hygro-DEST (w117-1) | ([Campeau *et al*, 2009](#_ENREF_1)) | Addgene plasmid #17454 |
| pLenti-CMV-Hygro-DEST (w117-1)-Blos1-WT | This Paper | N/A |
| pLenti-CMV-Hygro-DEST (w117-1)-Blos1-G449T | This Paper | N/A |
| psPAX2 | Trono lab | Addgene plasmid #12260 |
| pMD2.G | Trono lab | Addgene plasmid #12259 |

**References**

Campeau E, Ruhl VE, Rodier F, Smith CL, Rahmberg BL, Fuss JO, Campisi J, Yaswen P, Cooper PK, Kaufman PD (2009) A versatile viral system for expression and depletion of proteins in mammalian cells. *PLoS One* 4: e6529

Dubin MJ, Bowler C, Benvenuto G (2008) A modified Gateway cloning strategy for overexpressing tagged proteins in plants. *Plant Methods* 4: 3

Sanjana NE, Shalem O, Zhang F (2014) Improved vectors and genome-wide libraries for CRISPR screening. *Nat Methods* 11: 783-784
