# Supplementary material for: Brucella activates the host RIDD pathway to subvert BLOS1-directed immune defense
Source: eLife. 2022 May 19;11:e73625. doi: 10.7554/eLife.73625 (PMC9119680; doi:10.7554/eLife.73625)
Supplement: Supplementary file 2. — Primers used in this work. [file elife-73625-supp2.docx]

| **Table S3. Primers used in this work.** | | |  |
| --- | --- | --- | --- |
| Primer | 5'-3' Sequence | Source |  |
| Gapdh qPCR Primers FWD | AACAGCAACTCCCACTCTTC | This Paper |  |
| Gapdh qPCR Primers REV | CCTGTTGCTGTAGCCGTATT | This Paper |  |
| Blos1 qPCR FWD | GGCCTACATGAACCAGAGAAA | This Paper |  |
| Blos1 qPCR REV | AGTTCTCCACCATTCCAATCC | This Paper |  |
| Cd300lf qPCR FWD | GCATGTTTCCAACGCTGACT | This Paper |  |
| Cd300lf qPCR REV | GATGACTGGGAGGAGCACAC | This Paper |  |
| Diras2 qPCR FWD | GGACATTGCACTGGGGGT | This Paper |  |
| Diras2 qPCR REV | GAACTCCAGCTACTGACCAGG | This Paper |  |
| Txnip qPCR FWD | AGGCCTCATGATCACCATCT | This Paper |  |
| Txnip qPCR REV | GGTCTCAGCAGTGCAAACAG | This Paper |  |
| Blos1 BamHI FWD | AAAAGGATCCCTGTCCCGCCTGCTCAAAGAA | This Paper |  |
| Blos1 NotI REV | AAAAGCGGCCGCGGATGGTGCAGACTGCAG | This Paper |  |
| Blos1 g449t sense | GGATGGTGCAGACTGAAGCTGCCCTTTGTAG | This Paper |  |
| Blos1 g449t antisense | CTACAAAGGGCAGCTTCAGTCTGCACCATCC | This Paper |  |
| Blos1 gRNA primer 4 FWD | CACCGGAGGTGATCCACCAACGCTT | This Paper |  |
| Blos1 gRNA primer 4 REV | AAACAAGCGTTGGTGGATCACCTCC | This Paper |  |
| Blos1 gDNA FWD | CTGCAGCTGTCGCCCCCATCAGCG | This Paper |  |
| Blos1 gDNA REV | CCATTGCCCAAAGTAGTGGA | This Paper |  |
| Blos1 nested sequence gDNA | CCACAAAGTTACATTAGGGG | This Paper |  |
| Xbp (spliced) | GAGTCCGCAGCAGGTG | This Paper |  |
| Xbp1u Fwd | TTCCCATGGACTCTGACACTG | This Paper |  |
| Xbp1u Rev | GGTAGACCTCTGGGAGTTCTT | This Paper |  |
| U6 FWD | GCTTCGGCAGCACATATACTA | This Paper |  |
| U6 REV | CGAATTTGCGTGTCATCCTTG | This Paper |  |
| hsa-miR-17-5p Sense | TGCGCCAAAGTGCTTACAGTGCA | ([Song *et al*, 2020](#_ENREF_1)) | |
| hsa-miR-17-5p Antisense | CCAGTGCAGGGTCCGAGGTATT | ([Song *et al.*, 2020](#_ENREF_1)) |  |

References:

Song J, Liu Y, Wang T, Li B, Zhang S (2020) MiR-17-5p promotes cellular proliferation and invasiveness by targeting RUNX3 in gastric cancer. *Biomed Pharmacother* 128: 110246
