# Supplementary material for: Brucella activates the host RIDD pathway to subvert BLOS1-directed immune defense
Source: eLife. 2022 May 19;11:e73625. doi: 10.7554/eLife.73625 (PMC9119680; doi:10.7554/eLife.73625)
Supplement: Source data 1. [file elife-73625-data1.pdf]

Fig. 1Q and S

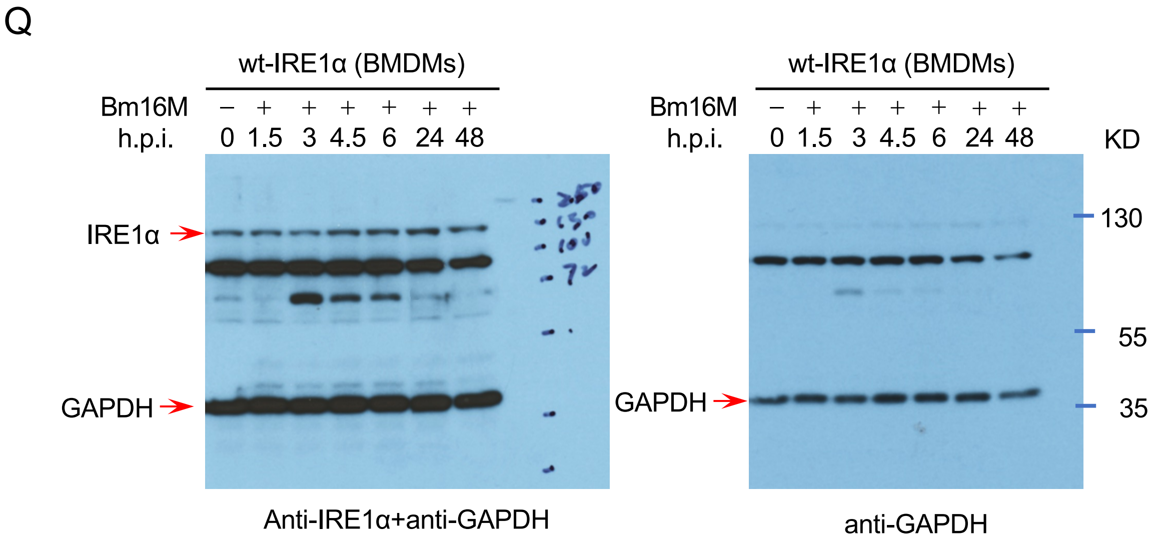

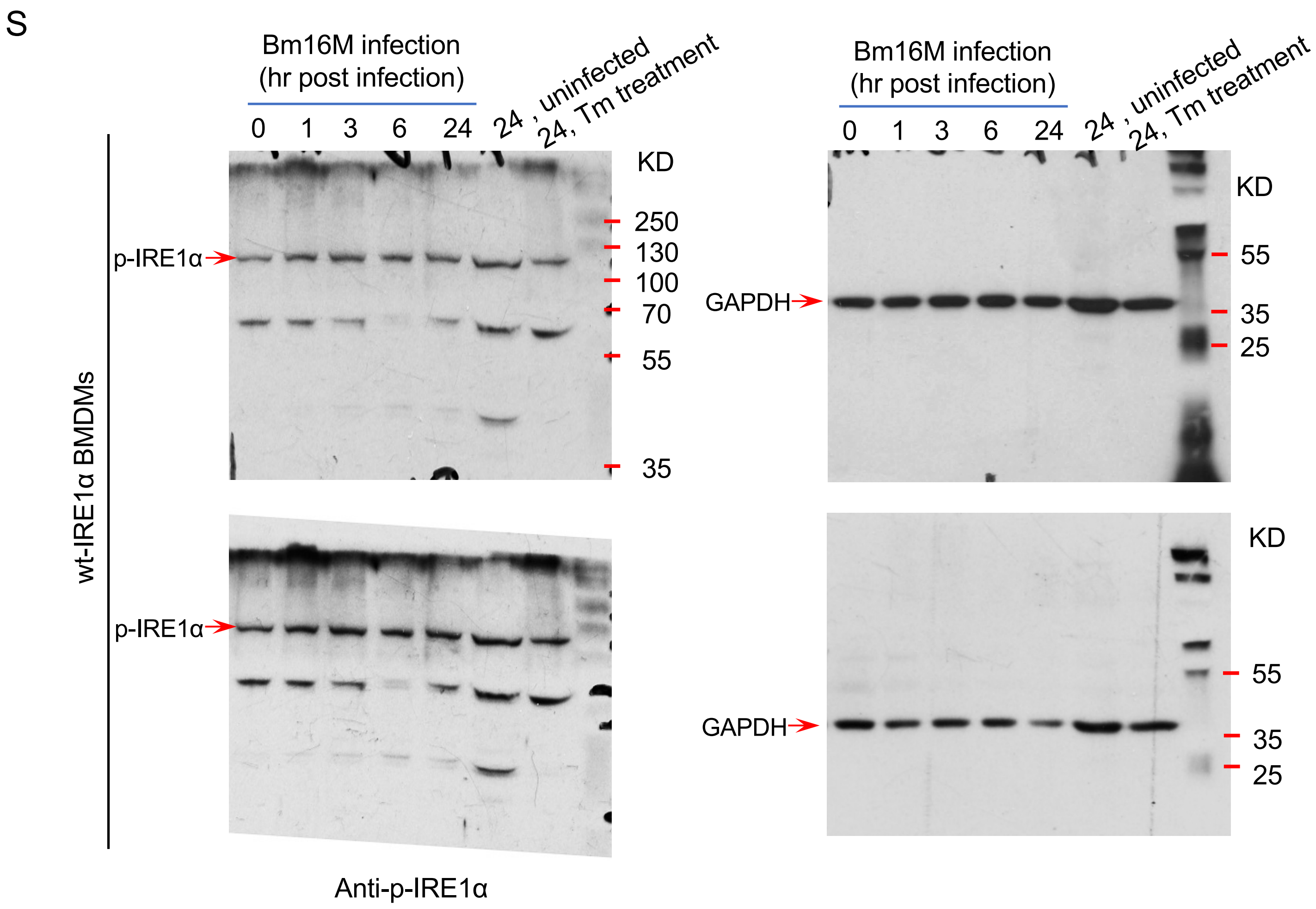

Target proteins are indicated by red arrows

Fig. 4A, C and G

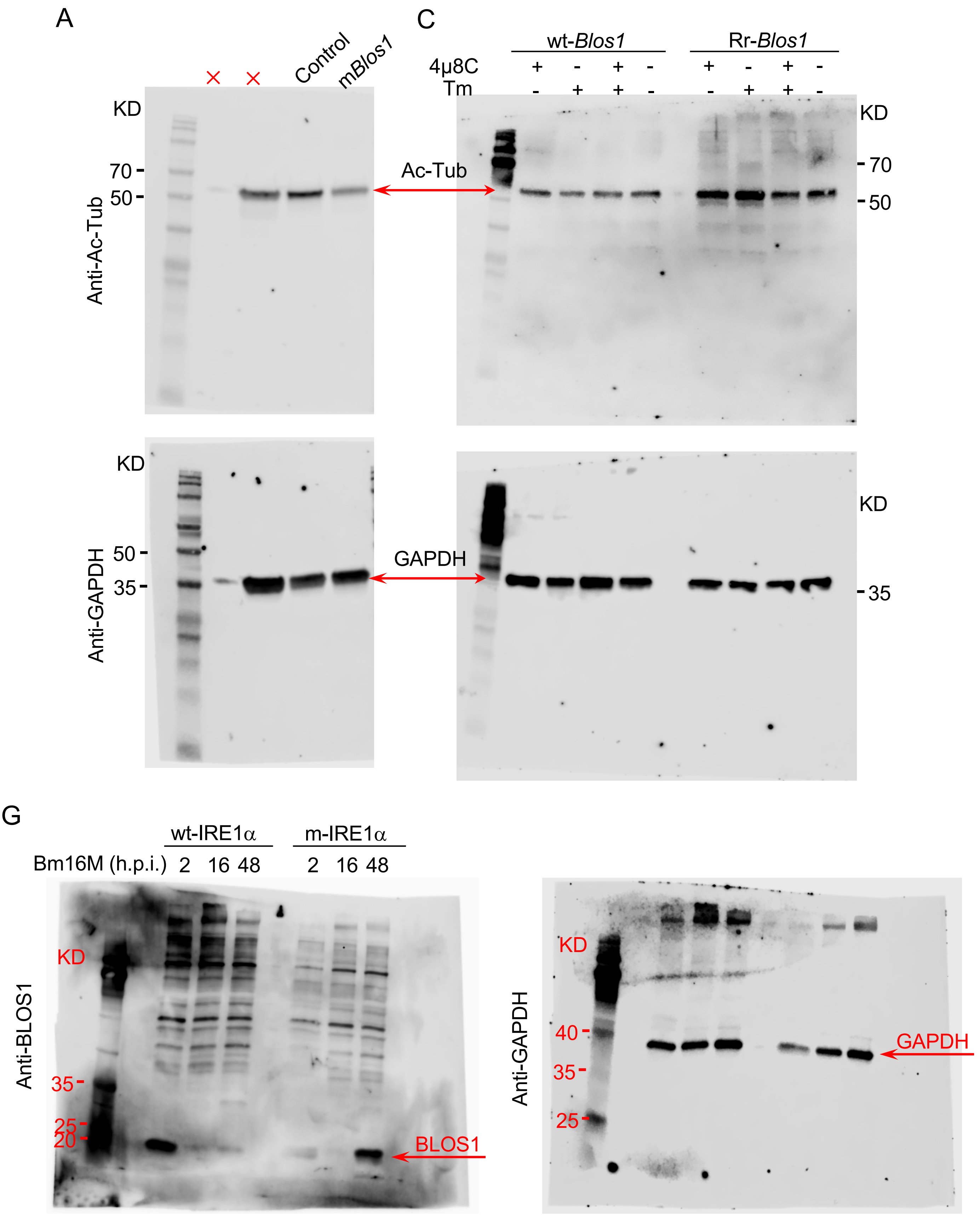

Target proteins are indicated by red arrows

Fig. 7A

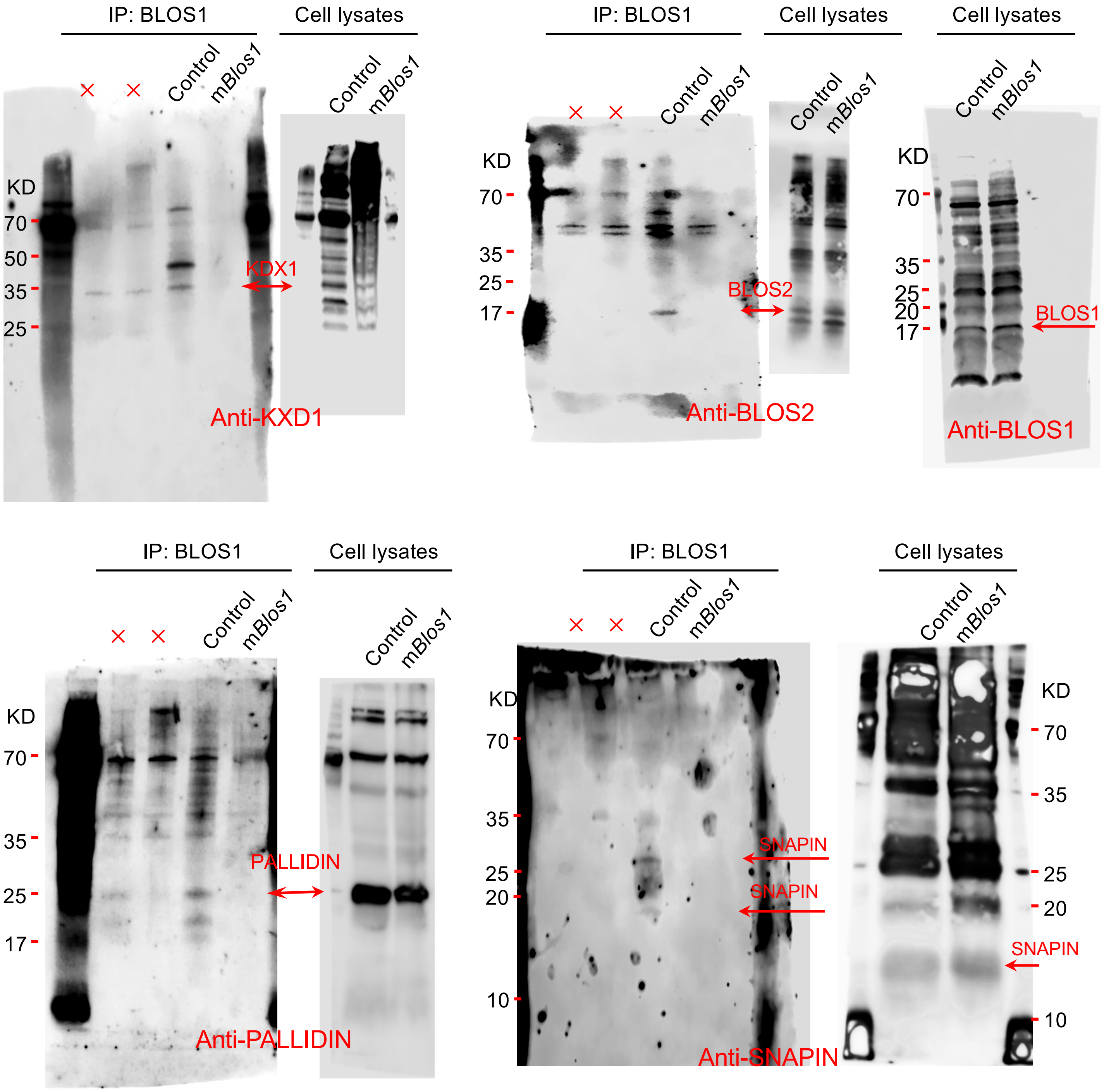

Target proteins are indicated by red arrows. SNAPIN antibody (Proteintech, Cat #: 10055-1-AP) can recognize the 15-18 KD (monomer) and 30-36 KD (dimer) forms of SNAPIN.

Fig. 7C, G

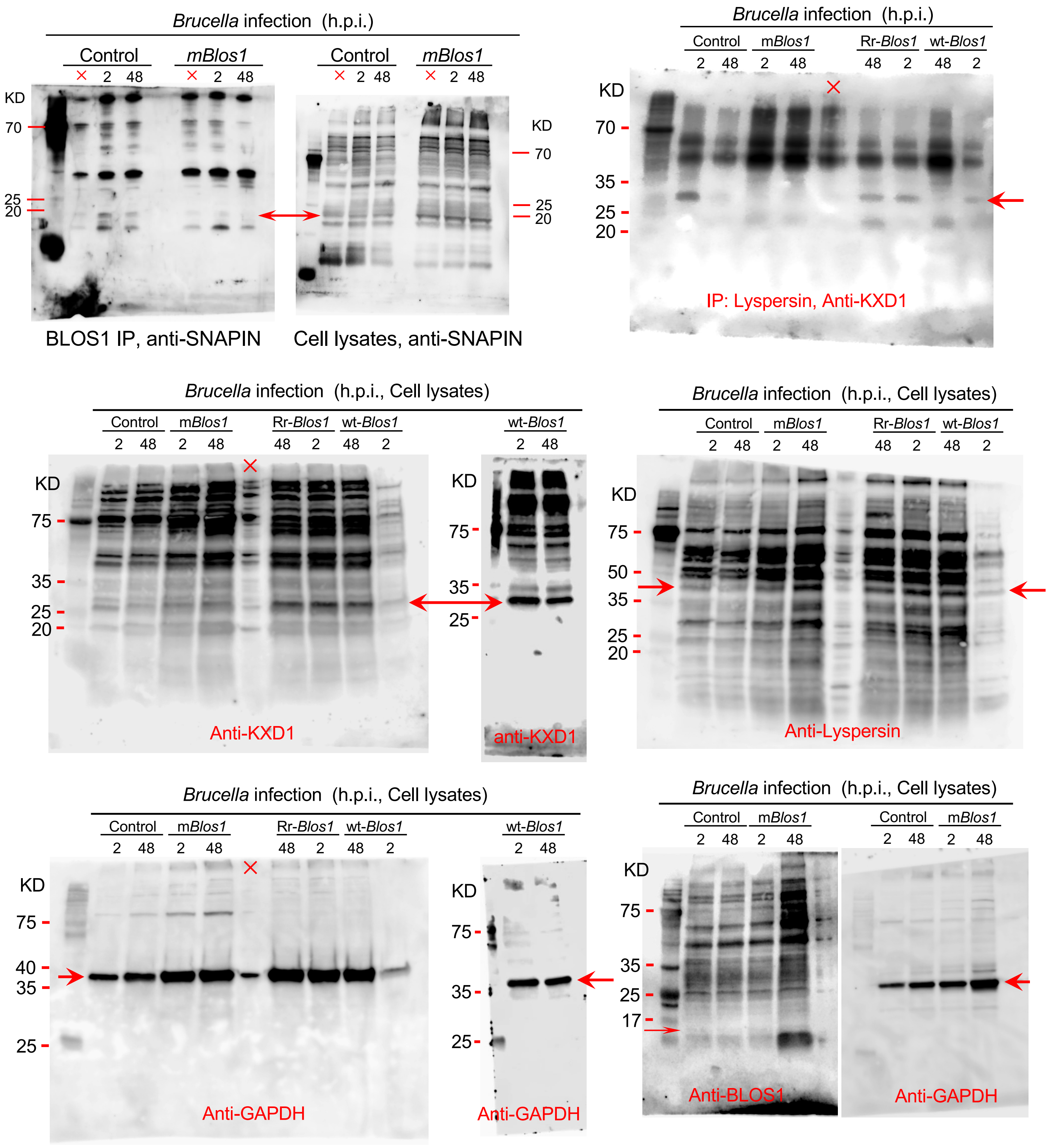

Target proteins are indicated by red arrows

Figure 1—figure supplement 1B

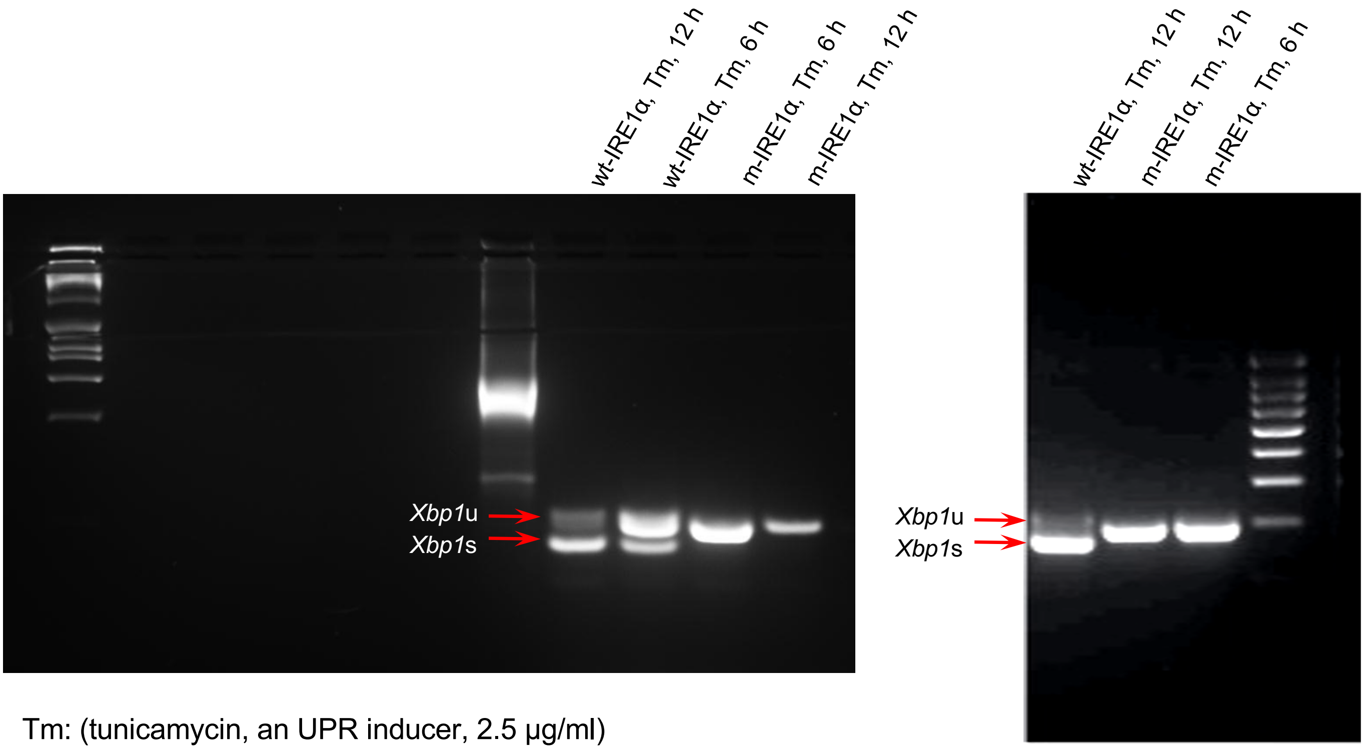

Tm: (tunicamycin, an UPR inducer, 2.5 μg/ml)

Figure 1—figure supplement 2A, C

A

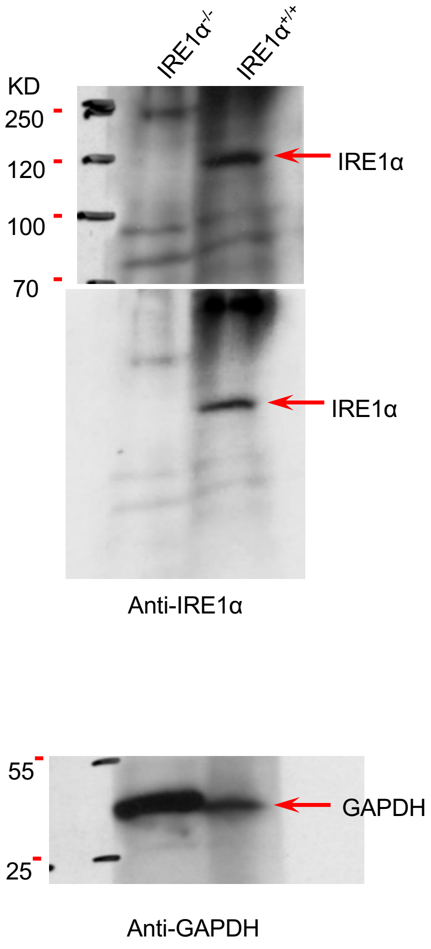

C

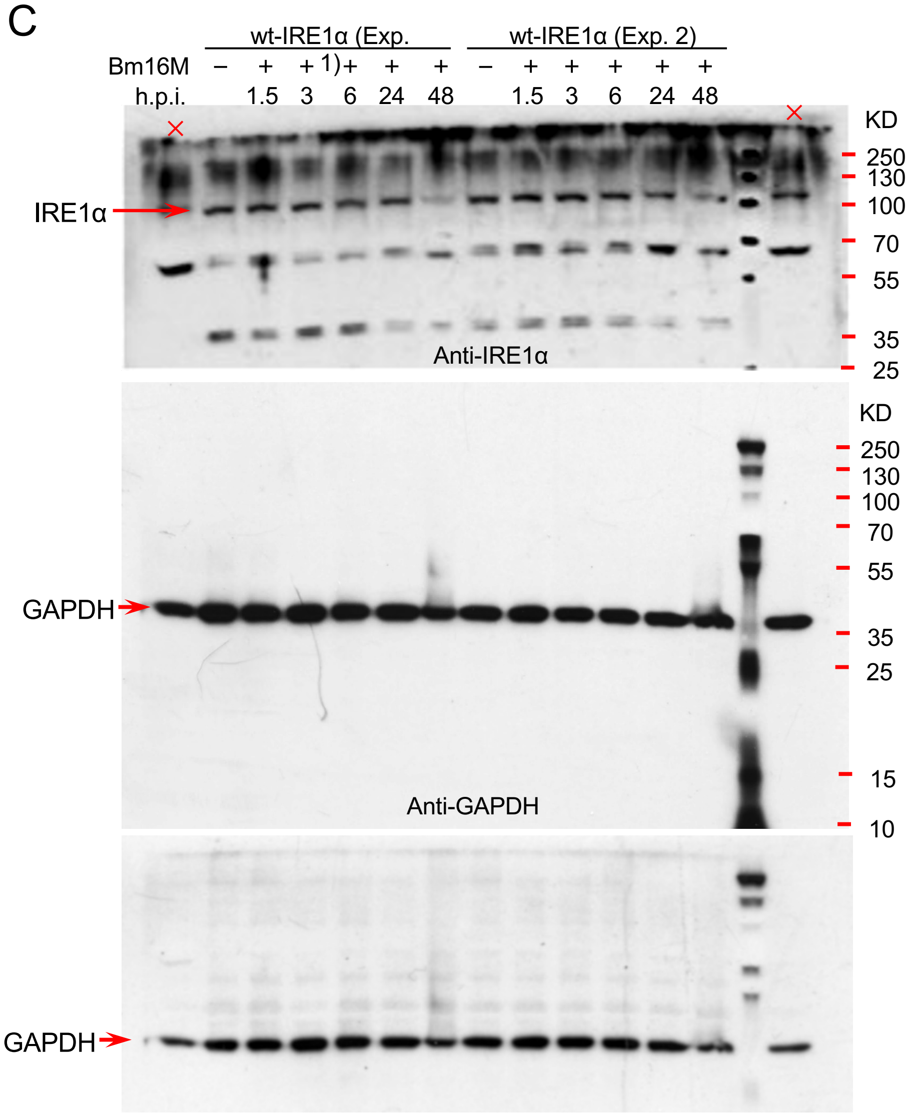

Target proteins are indicated by red arrows

Figure 1—figure supplement 2D, E

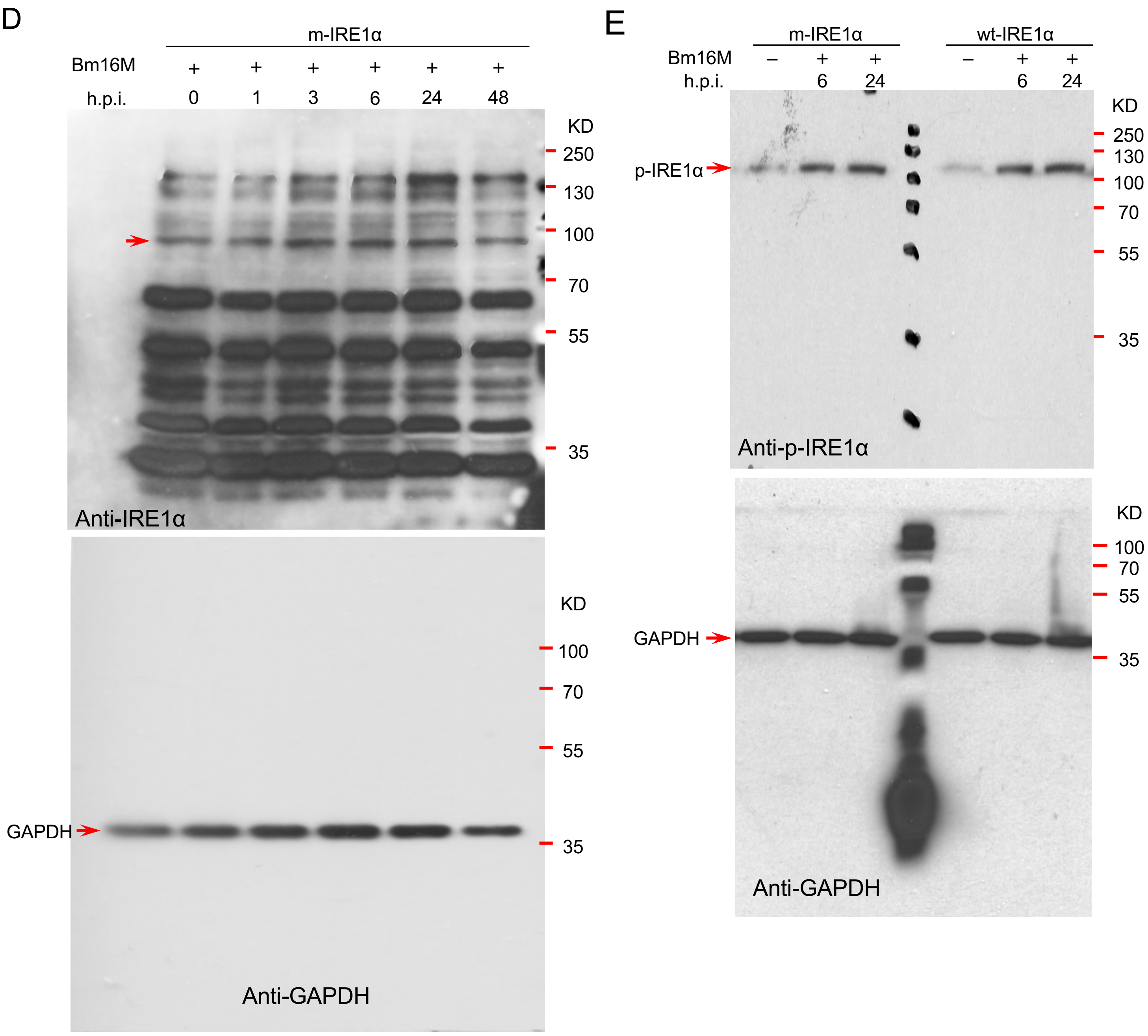

Figure 3—figure supplement 1A

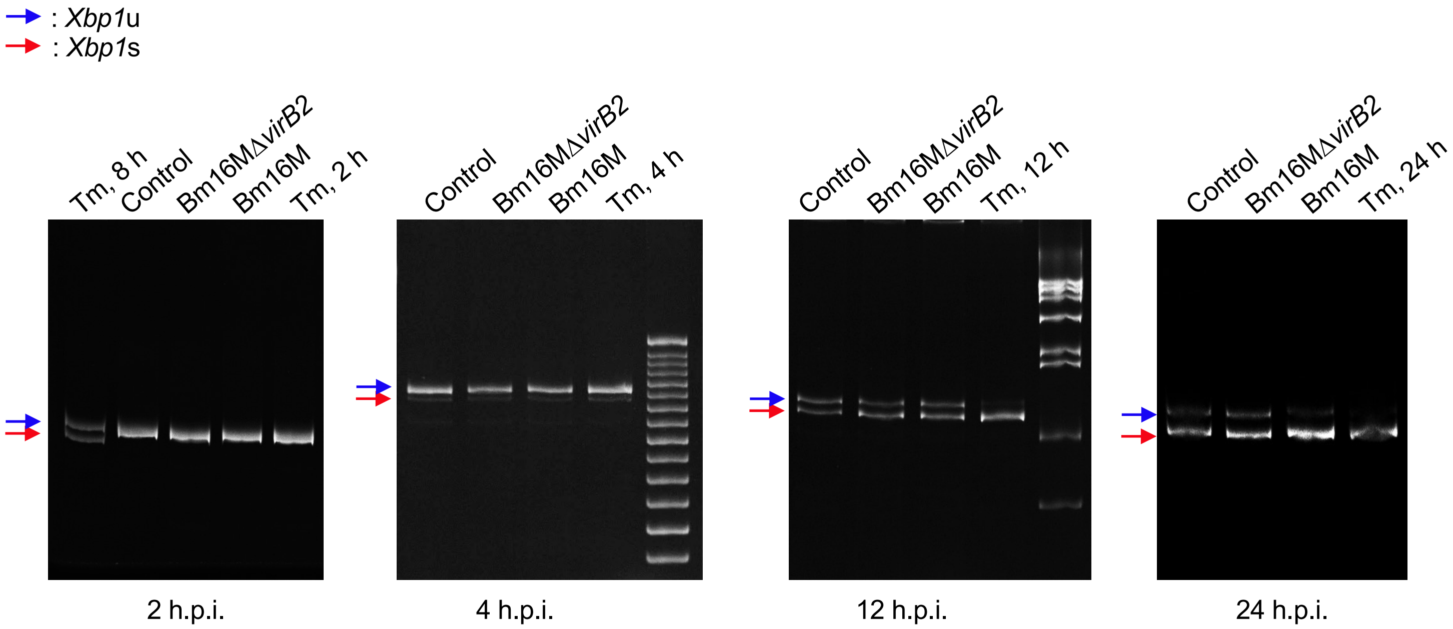

h.p.i.: hours post infection
